# Multimaterial decomposition in dual-energy CT for characterization of clots from acute ischemic stroke patients

**DOI:** 10.1186/s41747-024-00443-3

**Published:** 2024-04-05

**Authors:** Melina Gassenhuber, Maximilian E. Lochschmidt, Johannes Hammel, Tobias Boeckh-Behrens, Benno Ikenberg, Silke Wunderlich, Friederike Liesche-Starnecker, Jürgen Schlegel, Franz Pfeiffer, Marcus R. Makowski, Claus Zimmer, Isabelle Riederer, Daniela Pfeiffer

**Affiliations:** 1 Department of Diagnostic and Interventional Radiology, School of Medicine, Klinikum Rechts der Isar, Technical University of Munich, Munich, 81675 Germany; 2 Chair of Biomedical Physics, Department of Physics, School of Natural Sciences, Technical University of Munich, 85748 Garching, Germany; 3 Munich Institute of Biomedical Engineering, Technical University of Munich, 85748 Garching, Germany; 4 Department of Diagnostic and Interventional Neuroradiology, School of Medicine, Klinikum Rechts der Isar, Technical University of Munich, Munich, 81675 Germany; 5 Department of Neurology, School of Medicine, Klinikum Rechts der Isar, Technical University of Munich, Munich, 81675 Germany; 6 Pathology, Medical Faculty, University of Augsburg, 86150 Augsburg, Germany; 7 Department of Neuropathology, School of Medicine, Klinikum Rechts der Isar, Technical University of Munich, Munich, 81675 Germany; 8 Institute for Advanced Study, Technical University of Munich, 85748 Garching, Germany

**Keywords:** Blood coagulation, Ischemic stroke, Thrombectomy, Thrombosis, Tomography (x-ray computed)

## Abstract

**Background:**

Nowadays, there is no method to quantitatively characterize the material composition of acute ischemic stroke thrombi prior to intervention, but dual-energy CT (DE-CT) offers imaging-based multimaterial decomposition. We retrospectively investigated the material composition of thrombi *ex vivo* using DE-CT with histological analysis as a reference.

**Methods:**

Clots of 70 patients with acute ischemic stroke were extracted by mechanical thrombectomy and scanned *ex vivo* in formalin-filled tubes with DE-CT. Multimaterial decomposition in the three components, *i.e.*, red blood cells (RBC), white blood cells (WBC), and fibrin/platelets (F/P), was performed and compared to histology (hematoxylin/eosin staining) as reference. Attenuation and effective *Z* values were assessed, and histological composition was compared to stroke etiology according to the Trial of ORG 10172 in Acute Stroke Treatment (TOAST) criteria.

**Results:**

Histological and imaging analysis showed the following correlation coefficients for RBC (*r* = 0.527, *p* < 0.001), WBC (*r* = 0.305, *p* = 0.020), and F/P (*r* = 0.525, *p* < 0.001). RBC-rich thrombi presented higher clot attenuation in Hounsfield units than F/P-rich thrombi (51 HU *versus* 42 HU, *p* < 0.01). In histological analysis, cardioembolic clots showed less RBC (40% *versus* 56%, *p* = 0.053) and more F/P (53% *versus* 36%, *p* = 0.024), similar to cryptogenic clots containing less RBC (34% *versus* 56%, *p* = 0.006) and more F/P (58% *versus* 36%, *p* = 0.003) than non-cardioembolic strokes. No difference was assessed for the mean WBC portions in all TOAST groups.

**Conclusions:**

DE-CT has the potential to quantitatively characterize the material composition of ischemic stroke thrombi.

**Relevance statement:**

Using DE-CT, the composition of ischemic stroke thrombi can be determined. Knowledge of histological composition prior to intervention offers the opportunity to define personalized treatment strategies for each patient to accomplish faster recanalization and better clinical outcomes.

**Key points:**

• Acute ischemic stroke clots present different recanalization success according to histological composition.

• Currently, no method can determine clot composition prior to intervention.

• DE-CT allows quantitative material decomposition of thrombi *ex vivo* in red blood cells, white blood cells, and fibrin/platelets.

• Histological clot composition differs between stroke etiology.

• Insights into the histological composition *in situ* offer personalized treatment strategies.

**Graphical Abstract:**

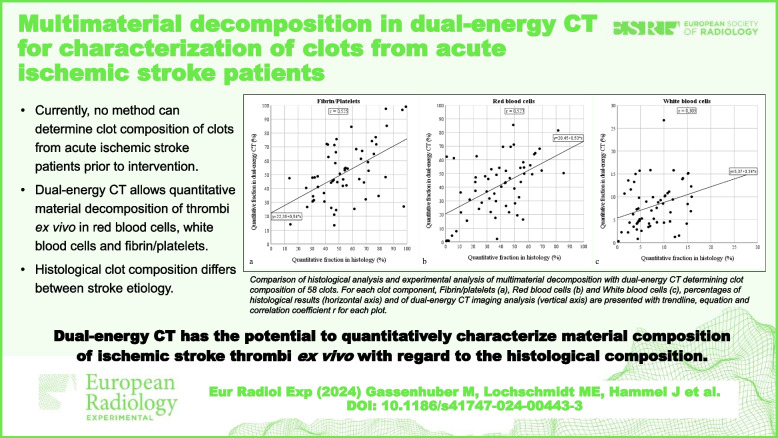

## Background

Acute ischemic stroke (AIS) is a common vascular disease associated with high morbidity and mortality [1]. Endovascular thrombectomy of large vessel occlusions and following histological analysis of extracted thrombus material allows characterization of AIS clots *ex vivo* and offers potential findings according to stroke etiology.

AIS thrombi consist of three main components, *i.e.*, red blood cells (RBC), white blood cells (WBC), and fibrin/platelets (F/P), and can be determined histologically using hematoxylin and eosin staining [2]. Previous studies showed associations between histological clot composition and etiology and present the potential to differentiate especially between cardioembolic and non-cardioembolic strokes [3–6]. Therefore, main stroke causes according to Trial of ORG 10172 in acute stroke treatment (TOAST) criteria [7] show differences in mean percentages of main clot components. Nevertheless, findings on the impact of histological clot composition on pathogenesis remain controversial [8, 9].

Besides, clots seem to present different treatment characteristics and recanalization success according to their histological composition and etiology [2, 10–13]. Component fractions may have an impact on treatment strategy and knowledge of exact material decomposition may be helpful prior to endovascular therapy to achieve better treatment success, higher recanalization rates, and more favorable clinical outcomes [14–16].

To date, there is no quantitative method for pre-interventional clot characterization is available that allows material decomposition without histological analysis. Initial computed tomography (CT) images of AIS patients show a positive association between attenuation in intracranial clots and RBC fraction [3]. The presence of a hyperdense artery sign and thrombus attenuation are related to stroke etiology [17] and can be detected in non-contrast CT. Besides, thrombus permeability in admission CT before and after release of contrast agent is associated with higher F/P and WBC fractions and is related to cardioembolic strokes [18].

Knowledge of the histological composition of intracranial clots could potentially help guide the choice between different treatment devices and may offer the opportunity of faster and more efficient recanalization of AIS. Dual-energy CT (DE-CT) presents information on attenuation of an object at two different energy levels. As the overall attenuation values in CT depend on the scanned material but also the energy level, DE-CT offers the possibility to quantitatively determine the specific sample material [19, 20].

In this study, we present a quantitative multimaterial decomposition approach for *ex vivo* thrombi with different stroke etiologies using DE-CT, with histological analysis of clot composition as reference.

## Methods

### Study patients

This retrospective study was approved by our local ethics committee and informed consent of patients was waived due to the retrospective design. A total of 79 AIS thrombi were collected by endovascular thrombectomy between June 2020 and April 2021 and received postinterventional DE-CT imaging and histological analysis. Finally, 70 thrombi were included in this study. Four clots with inadequate image quality, one patient with sinus venous thrombosis, and 1 patient with iatrogenic AIS after interventional treatment of a subarachnoid hemorrhage were excluded. In addition, for 3 patients with multiple events, only the first AIS event was included, subsequent events in the same patients were excluded.

### Clot collection and clinical data

**Fig. 1:**
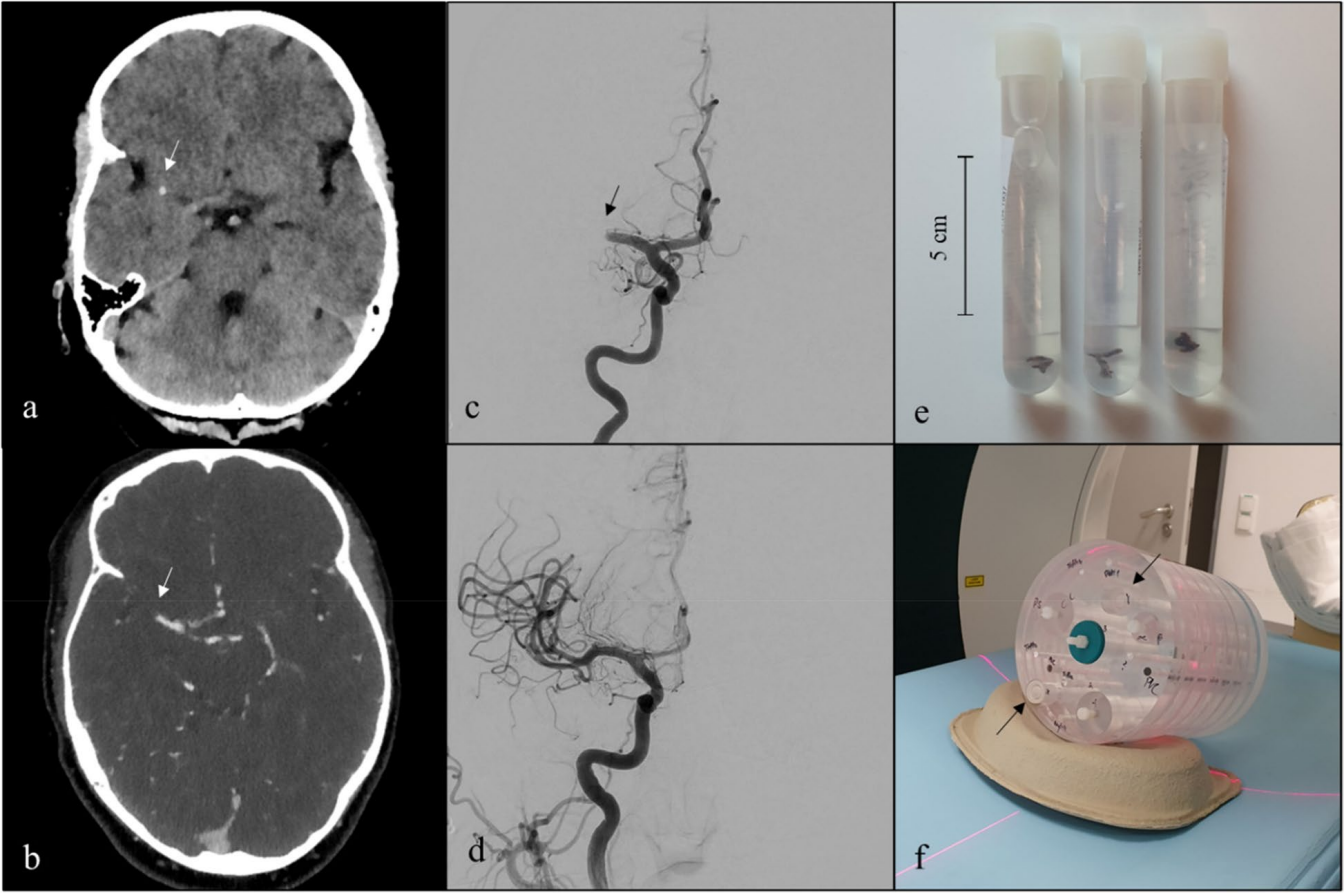
Initial CT and angiographic series of a stroke patient and experimental set-up of clot scans. **a**–**d** Examinations of a 47-year-old woman with AIS. **a** Initial non-enhanced cerebral CT image in axial view with hyperdense artery sign in the right middle cerebral artery (white arrow). **b** Initial cerebral CT angiography in axial view with stop of contrast agent in the right middle cerebral artery in the M1 segment where the thrombus is located (white arrow). **c**, **d** Angiographic series of the patient in frontal view before (**c**) and after (**d**) mechanical thrombectomy. **e** Three examples of different formalin-fixed thrombi after thrombectomy. **f** Two thrombi inside the phantom model (black arrows). AIS, Acute ischemic stroke; CT, Computed tomography

All patients underwent endovascular recanalization therapy within clinical routine. According to institutional practice and depending on individual patient characteristics, interventional therapy was performed with a combination of distal access aspiration catheter, stent retriever, and balloon guide catheter. Extracted clot material was immediately fixed in phosphate-buffered 3.5–3.7% formalin. The experimental setup of DE-CT imaging is shown in Fig. 1.

Data of demographic, clinical, and interventional parameters were collected. In accordance with the international TOAST classification [7], the most likely cause of AIS was determined individually using clinical and diagnostic information, including cerebral CT imaging, MRI, transcranial and extracranial duplex sonography, transthoracic or transesophageal echocardiography, long-term electrocardiography, and coagulation tests.

### Imaging protocol and scan assessment

DE-CT scan was performed using a dual-layer CT (IQon spectral CT, Philips Healthcare, Best, The Netherlands) with tube voltage of 120 kVp, exposure of 500 mAs, rotation time of 0.75 s, pitch of 0.328 s and axial slice thickness of 0.8 mm. Spectral level 2 and Brain Sharp (UC) filter were used for scans. The volumetric CT dose index-CTDI_vol_ was 85.9 mGy for all scans and the mean dose length product-DLP was 215 mGy*cm. Extracted thrombus material stored in formalin-filled tubes was scanned inside a phantom model to accomplish equal scan conditions. The phantom model was tilted for scanning so that all thrombus material was located on the bottom of the tubes. Time between thrombus extraction and DE-CT scan was determined as storage time. Post-processing and imaging analysis were performed using a Philips internal software. Conventional CT and virtual monoenergetic images at 50 keV and 200 keV were generated from spectral data sets for later analysis.

With DE-CT data sets a multimaterial decomposition was performed. In general, the method for multimaterial decomposition relies on the fact that for two different modalities, which are commonly sensitive to three of the five materials to be analyzed and the remaining two materials can only be co-determined by one of these modalities, a mathematical relationship can be established. This relationship is described in such a way that the sum of all volume fractions of that modality, which is sensitive to all five materials, can be equated with a modified representation of the other modality.

For this, the result of the second modality is multiplied by a correction factor variable and then the missing two volume fractions are added. Letting the absorption coefficients of the base material and the fact that for both modalities the sum of all volume parts is 1, flow in, it is possible to determine a function which has a global minimum at 0. A global optimizer was then used to determine the correction factor and the missing two materials in such a way that the function takes its minimum sufficient close to 0. In our specific case, the first modality, which is sensitive to all five materials RBC, WBC, F/P, iodine, and formalin, is the DE-CT and the second modality is the histological finding, which only includes the proportions of RBC, WBC, and F/P. This then enables a quantitative differentiation of RBC, WBC, F/P, iodinated contrast medium, and formalin to determine the clot composition [21].

All 70 clots were previously reported in a prior work describing the technical development of the method [21], while this study focused on the clinical correlations. Twelve thrombi were used for the optimization of the algorithm and were excluded for statistical analysis that considered 58 of 70 clots. The selection of appropriate thrombi for the optimization of base materials is crucial, as only a successful optimization of the base materials can ensure a successful decomposition of additional thrombi. For this purpose, arbitrary samples cannot be utilized. Collectively, they must satisfy several criteria. Primarily, only those samples were employed that vary in their composition in such a manner that the entire space, which is confined by the maximum and minimum feasible values of RBC, WBC, and F/P, is covered as uniformly as possible. Only in this manner is the successful optimization of the base materials valid for additional samples, which are situated in their composition in this confined space. Moreover, samples were utilized which have been extensively rinsed with formalin and hence no diffused iodine can be detected in the thrombus. This is significant because the optimization of the base materials does not rectify the iodine. Considering all criteria, it was found that the optimization for 12 samples converges adequately well on the one hand and the optimization time is merely a few minutes on the other hand. Consequently, 12 samples were chosen which satisfy all the mentioned criteria.

**Fig. 2:**
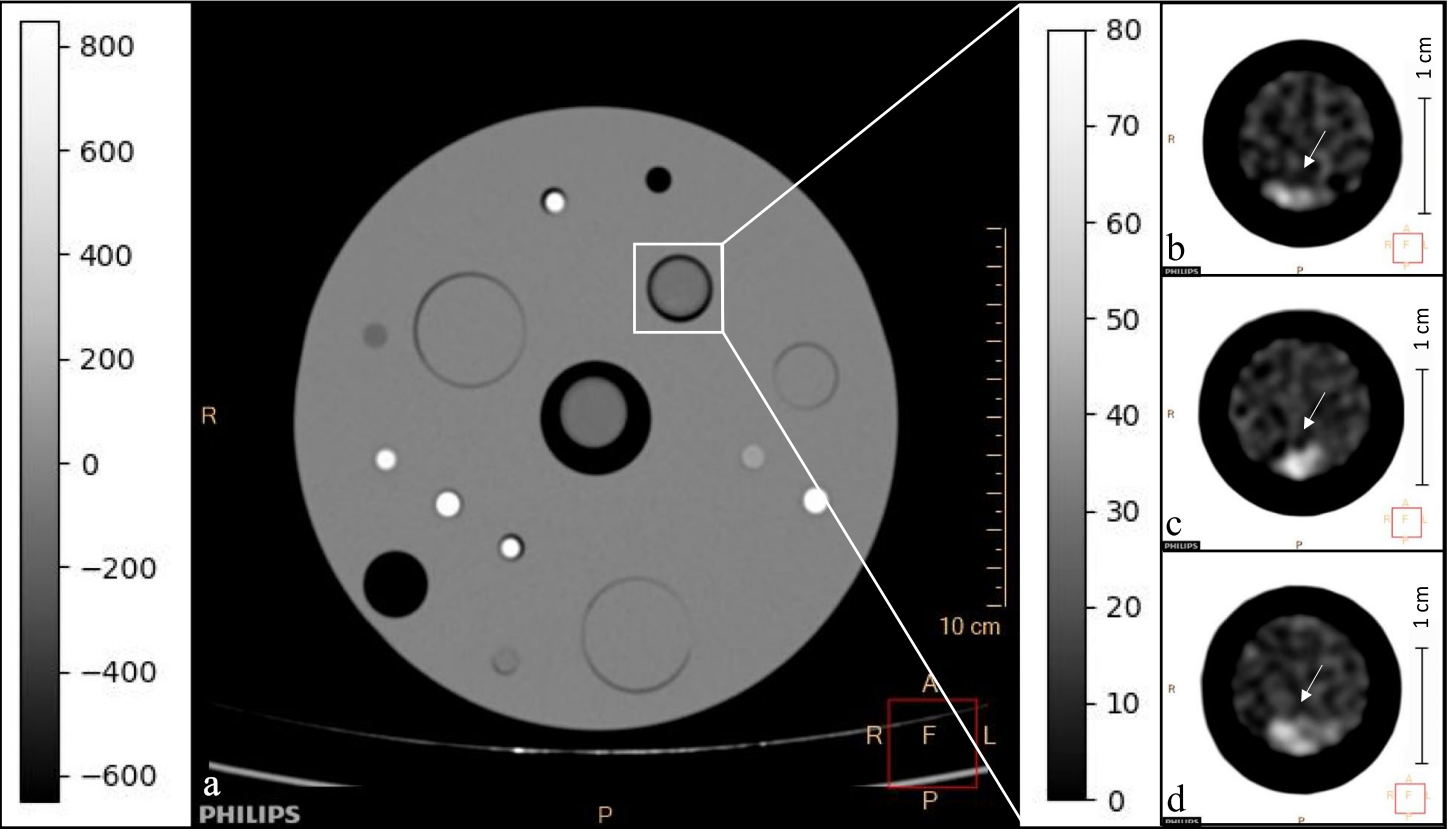
Examples of attenuation images in DE-CT.** a** DE-CT scan in the axial view of a formalin-fixed clot in a tube inside a phantom model, scale bar is attached on the left. Three different examples of thrombi scanned in DE-CT in axial view (white arrows): F/P-rich thrombus (F/P 63%, 42 HU) (**b**), RBC-rich thrombus (RBC 63%, 64 HU) (**c**), and mixed thrombus (F/P 39%, RBC 56%, 59 HU) (**d**). DE-CT, Dual-energy computed tomography; F/P, Fibrin/platelets; HU, Hounsfield units; RBC, Red blood cells

Additionally, three regions of interest (ROIs), 1 mm^2^, 1.5 mm^2^, and 2 mm^2^, were drawn in different parts of thrombi in conventional CT to determine mean clot attenuation and effective *Z* values. Attenuation images in DE-CT of different clot examples according to clot component dominance are shown in Fig. 2.

### Histological analysis of thrombus material

After scanning thrombus material was transferred to 70% ethanol, embedded in paraffin, and cut into 2-µm slices. The processing of slices was completed with hematoxylin and eosin staining and slices were scanned at high resolution (40 ×) and digitally stored. Histological characterization into the main components RBC, WBC, and F/P was performed blind to clinical data. As it is impossible to differentiate fibrin and platelets with H&E staining, these areas were combined as F/P.

The percentage distribution of each component was assessed by using Orbit Image Analysis, a free open-source software using machine learning segmentation [22]. One representative slice of thrombus material was selected for analysis. First of all, only thrombus material was selected using the exclusion model, while the background of digitized slices was excluded. In cases of overlapping material or unfavorable staining, affected areas were excluded by using a second exclusion model. Subsequently, the three main clot components were defined by selecting between three to five different areas of each component. In this classification, the machine learning segmentation enables the differentiation between the three clot components and the quantitative analysis can be performed automatically with the percentage of clot composition.

### Statistical analysis

Descriptive statistics for demographic, clinical, and interventional parameters, as well as for histological and imaging analysis were evaluated. Nonparametric Kruskal–Wallis test was used to show differences between TOAST groups and results were presented with box plots. Shapiro–Wilk test was used for normal distribution analysis. Histological analysis was compared with multimaterial decomposition using Pearson’s correlation coefficient for RBC and F/P and Spearman’s rank correlation coefficient for WBC. Bland–Altman analysis was performed, and Bland–Altman plots are presented. Component percentages were correlated with CT attenuation and effective *Z* values using Spearman’s rank correlation coefficient. Group differences between CT parameters and clot composition were presented using nonparametric Kruskal–Wallis tests. Statistical analysis was performed with the SPSS software. Test results with *p* < 0.05 were considered as statistically significant.

## Results

### Demographics and clinical data

In total, 70 of the 79 patients met the inclusion criteria. Basic clinical data, occlusion site, and stroke etiology according to the TOAST criteria are summarized in Table 1.

**Table 1:** Clinical characteristics of all 70 patients in this study

Characteristic	Value
Patients, *n*	70
Age, years, mean ± standard deviation (range)	76 ± 12 (46–96)
Sex, *n* (men/women)	30/40
Localization of occlusion, *n* (%)	
Internal carotid artery/carotid-T	14 (20)
M1 segment of the middle cerebral artery	33 (47)
M2 segment of the middle cerebral artery	9 (13)
Combined internal carotid artery and M1 segment of the middle cerebral artery	8 (11)
A2 segment of the anterior cerebral artery	2 (3)
Vertebrobasilar	4 (6)
Stroke etiology (TOAST), *n* (%)	
Arterioembolic (TOAST-1)	6 (9)
Cardioembolic (TOAST-2)	33 (47)
Other determined cause (TOAST-4) (3 dissections, 1 in-stent thrombosis, 1 patent foramen ovale)	5 (7)
Cryptogenic (TOAST-5)	26 (37)

*TOAST* Trial of ORG 10172 in Acute Stroke Treatment

### Histological clot composition and correlation with stroke etiology

**Fig. 3:**
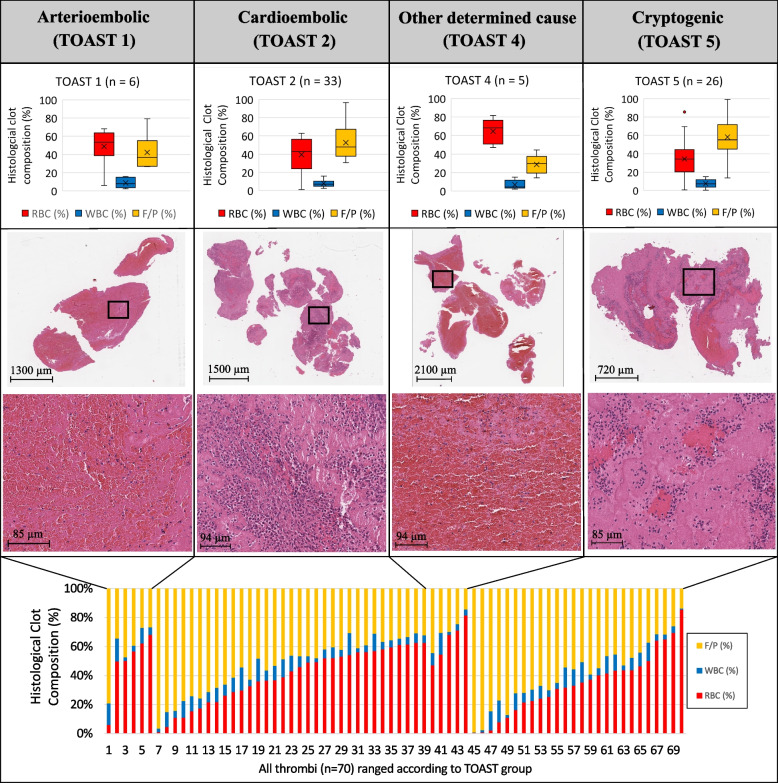
Overview of four groups of stroke etiology in this study according to the TOAST criteria. *First row*: group differences in clot composition, including the three main clot components RBC, WBC, and F/P, are shown in box plots and ranged according to the four TOAST groups included in this study, arterioembolic (TOAST-1), cardioembolic (TOAST-2), other determined cause (TOAST-4), and cryptogenic (TOAST-5) strokes. *Second and third rows*: a representative thrombus slice with hematoxylin and eosin staining for each TOAST group and magnification of the black squares below. With hematoxylin and eosin staining, RBC are presented in red, F/P are stained pink, and WBC are presented as little blue cells with nuclei. *Fourth row*: Graphical representation of histological clot composition including RBC, WBC, and F/P of each of the 70 thrombi included in this study ranged according to the TOAST group. F/P, Fibrin/platelets; RBC, Red blood cells; TOAST, Trial of ORG 10172 in Acute Stroke Treatment; WBC, White blood cells

All clots showed a heterogeneous pattern of the three main clot components RBC, WBC, and F/P. The quantitative thrombus composition of all 70 clots is summarized at the bottom of Fig. 3. The percentages (mean ± standard deviation in %) of RBC (40 ± 21), WBC (8 ± 4), and F/P (52 ± 20) were assessed. In addition, the clots were classified according to their component dominance in 13 RBC-rich (≥ 60% of RBC), 23 F/P-rich (≥ 60% of F/P), and 34 mixed thrombi.

The quantitative clot composition was analyzed according to stroke etiology and one representative clot slice for each stroke cause including magnification is shown in Fig. 3.

**Fig. 4:**
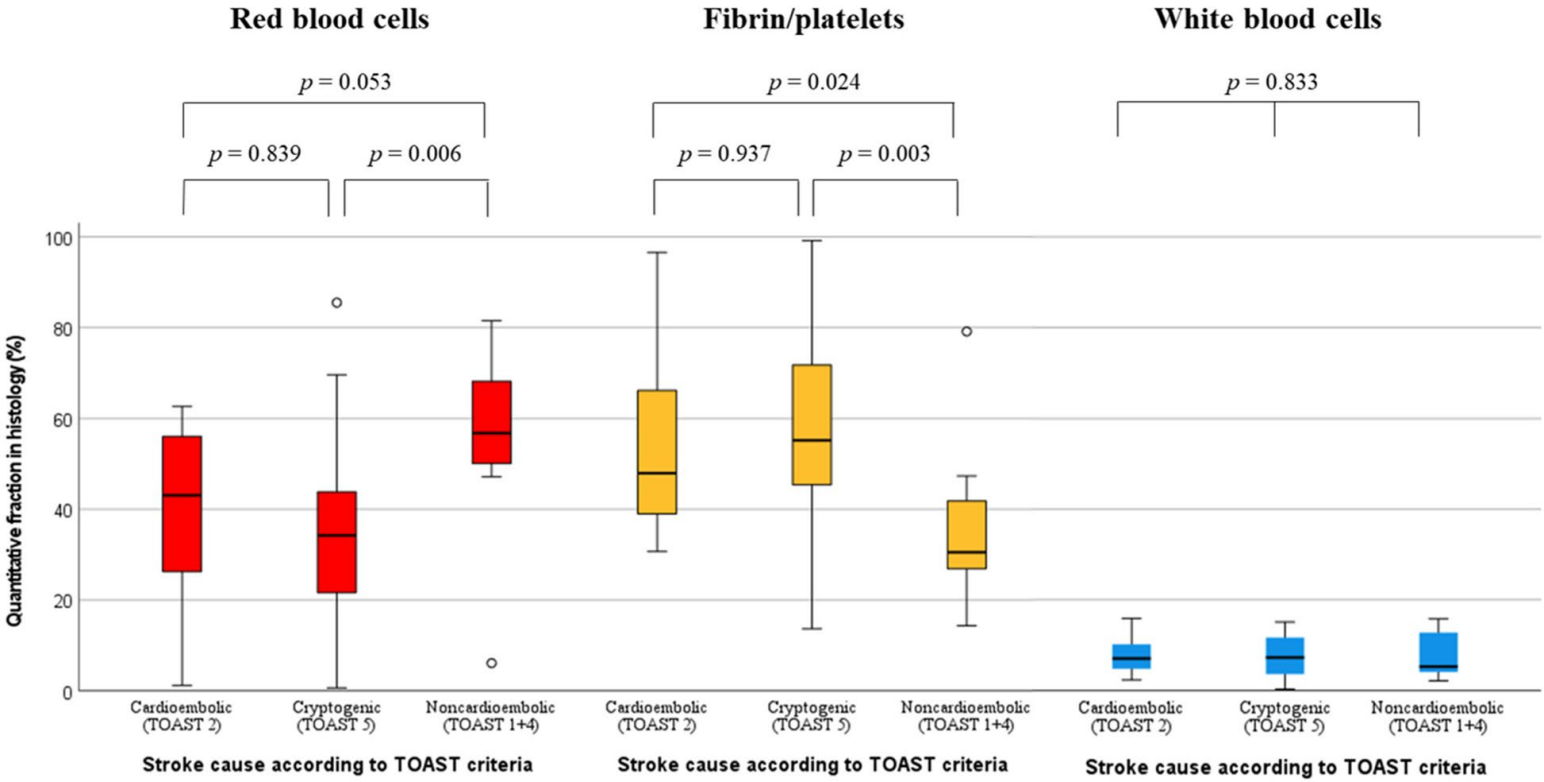
Difference in quantitative fraction in histology between cardioembolic, cryptogenic, and non-cardioembolic strokes. Mean percentages of each clot component, *i.e.*, red blood cells, fibrin/platelets, and white blood cells, are shown in box plots according to three TOAST groups: cardioembolic (TOAST-2), cryptogenic (TOAST-5), and non-cardioembolic strokes (TOAST-1 and TOAST-4). Arterioembolic (TOAST-1) and strokes of other determined causes (TOAST-4) are summarized as non-cardioembolic. Statistical analysis was performed by using the nonparametric Kruskal–Wallis test and *p* values are presented in the diagram. Cardioembolic clots showed fewer mean proportions of RBC (*p* = 0.053) and more F/P (*p* = 0.024) than non-cardioembolic strokes. Similarly, cryptogenic strokes contained less RBCs (*p* = 0.006) and higher fractions of F/P (*p* = 0.003) compared to non-cardioembolic. Comparison between cardioembolic and cryptogenic strokes presented similar histological clot composition for RBC (*p* = 0.839) and F/P (*p* = 0.937). Mean percentages of WBCs showed no difference (*p* = 0.833) in non-cardioembolic compared to cardioembolic and cryptogenic strokes. *TOAST* Trial of ORG 10172 in Acute Stroke Treatment

Clot composition was associated with stroke etiology according to TOAST classification. Arterioembolic (TOAST-1) and strokes of other determined cause (TOAST-4) were summarized as non-cardioembolic and were compared to cardioembolic (TOAST-2) and cryptogenic (TOAST-5) strokes. Cardioembolic clots showed less mean proportions of RBC (40% *versus* 56%, *p* = 0.053) and more F/P (53% *versus* 36%, *p* = 0.024) than non-cardioembolic strokes. Similarly, cryptogenic strokes contained less RBCs (34% *versus* 56%, *p* = 0.006) and higher fractions of F/P (58% *versus* 36%, *p* = 0.003) compared to non-cardioembolic. Mean percentages of WBCs showed no significant difference (*p* = 0.833) in non-cardioembolic (8%) compared to cardioembolic (8%) and cryptogenic (7%) strokes. Comparison between cardioembolic and cryptogenic strokes presented similar histological clot composition for RBC (40% *versus* 34%, *p* = 0.839) and F/P (53% *versus* 58%, *p* = 0.937). Differences in quantitative fraction in histology between cardioembolic, cryptogenic, and non-cardioembolic strokes are presented in Fig. 4 with attached statistical analysis.

### Imaging analysis of thrombi with dual-energy CT

**Fig. 5:**
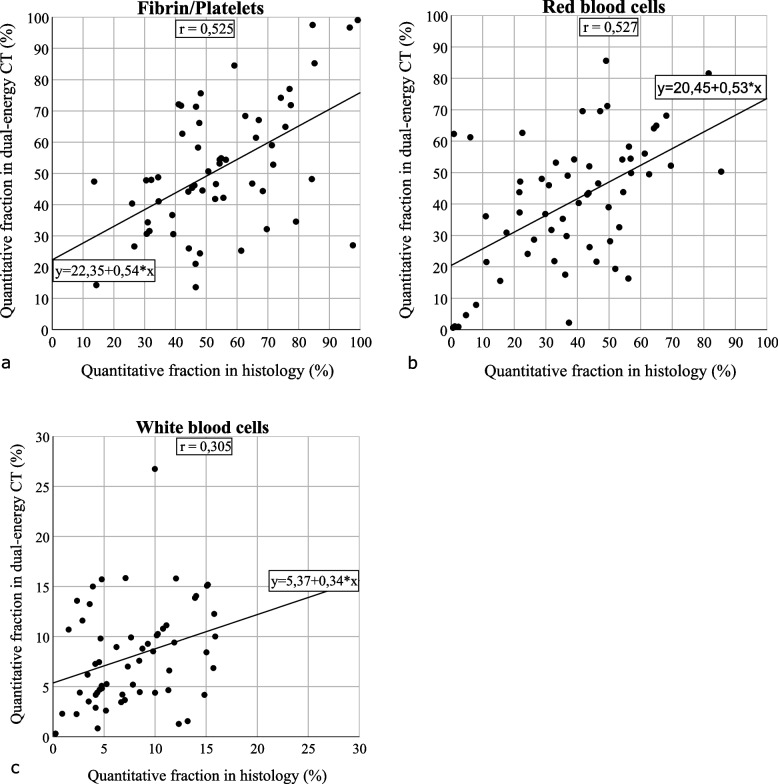
Comparison of histological analysis and experimental analysis of multimaterial decomposition with DE-CT determining clot composition. For this evaluation, 58 of the 70 clots were included, as 12 thrombi were already used for the optimization of the algorithm. For each clot component, F/P (**a**), RBC (**b**), and WBC (**c**), percentages of histological results are shown in the horizontal axis, and quantitative fractions of DE-CT imaging analysis are presented in the vertical axis. The trendline, equation, and correlation coefficient *r* are presented for each plot. DE-CT, Dual-energy computed tomography; F/P, Fibrin/platelets; RBC, red blood cells; WBC, White blood cells

**Fig. 6:**
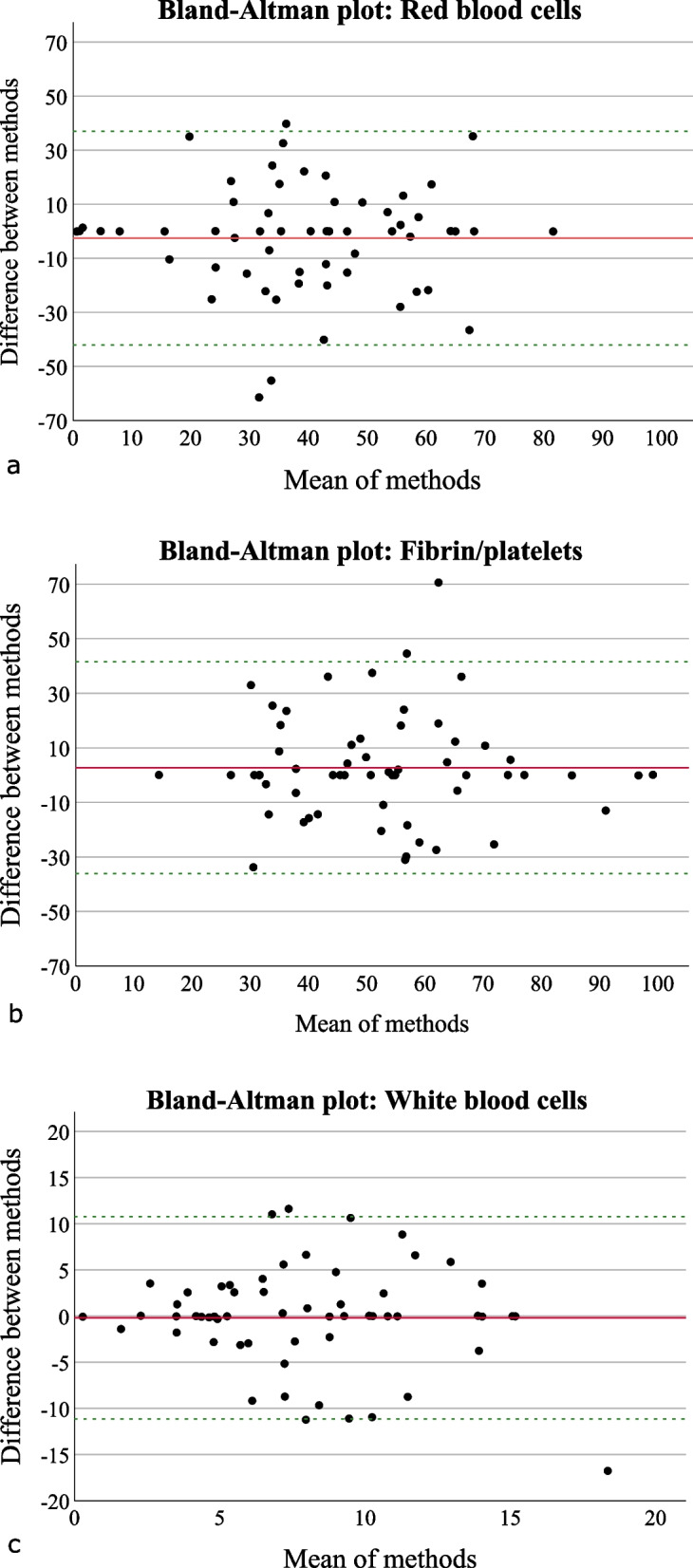
Bland–Altman plot for each clot component, red blood cells (**a**), fibrin/platelets (**b**), and white blood cells (**c**). In the diagram, the mean difference (red line) and standard deviation of the differences, +1.96 × standard deviation and -1.96 × standard deviation (green dotted line), are given

For evaluation of the multimaterial decomposition using DE-CT, 58 of the 70 clots were included in statistical analysis, as 12 thrombi were used for the optimization of the algorithm and could possibly interfere with statistical findings. Results of quantitative multimaterial clot decomposition and histological clot characteristics are shown for each of the 3 main clot components, F/P, RBC, and WBC in Fig. 5. For each plot trendline, equation and *r* values are given. Evaluating the accordance of both methods assessing the quantitative mixture of stroke thrombi, correlation coefficients were determined for each main component, RBC (*r* = 0.527, *p* < 0.001), WBC (*r* = 0.305, *p* = 0.020), and F/P (*r* = 0.525, *p* < 0.001). Bland–Altman analysis was performed afterwards, and Bland–Altman plots are presented in Fig. 6.

Further imaging analysis included all 70 clots. Descriptive values of storage time, clot attenuation in Hounsfield units (HU), and effective *Z* values as mean values of three ROIs in different parts of clot material in conventional CT are summarized in Table 2. For further imaging analysis, clots were divided into 3 groups according to component dominance in RBC-rich, F/P-rich, and mixed thrombi, and group tests using the nonparametric Kruskal–Wallis test were performed for detecting differences in mean storage time, HU, and effective *Z* values. The mean storage time of thrombi was about 49 h and presented a wide range between 1 and 173 h. This resulted in a relatively high heterogeneity of thrombus age after extraction, but mean storage time in hours showed no significant difference (*p* = 0.893) between RBC-rich (43), F/P-rich (48), and mixed thrombi (51). The mean clot attenuation showed a significant difference between the three groups according to component dominance (*p* = 0.006), and the Dunn-Bonferroni post hoc test has been performed. RBC-rich clots showed higher mean HU values in conventional CT than F/P-rich thrombi (51 *versus* 42, *p* = 0.005). The mean density value of mixed thrombi (47 HU) presented values between the two other groups but showed no significant difference between RBC-rich (*p* = 0.250) and F/P-rich (*p* = 0.160) groups. Additionally, there was no significant group difference in effective *Z* values (*p* = 0.544).

**Table 2:** DE-CT imaging characteristics of all 70 clots

Clot characteristics	All clots (*n* = 70)	RBC-rich (*n* = 13)	F/P-rich (*n* = 23)	Mixed (*n* = 34)
Storage time, h, mean ± standard deviation (range)	49 ± 38 (1−173)	43 ± 30 (6−93)	48 ± 30 (1−106)	51 ± 45 (4−173)
Attenuation, HU, mean ± standard deviation	46 ± 9	51 ± 10	42 ± 8	47 ± 9
Effective *Z* value, mean ± standard deviation	7.2 ± 0.1	7.1 ± 0.1	7.2 ± 0.1	7.2 ± 0.1

Mean storage time (*p* = 0.893) and effective *Z* values (*p* = 0.544) showed no significant group difference between RBC-rich, F/P-rich, and mixed thrombi using nonparametric Kruskal–Wallis. Mean attenuation showed only a significant difference between RBC-rich and F/P-rich thrombi (*p* < 0.01) in the Dunn-Bonferroni post hoc test. Mean attenuation of mixed thrombi showed no significant difference between RBC-rich (*p* = 0.250) and F/P-rich thrombi (*p* = 0.160). *F/P* Fibrin/platelets, *RBC* Red blood cells

Correlation analysis between clot attenuation and histological clot characteristics using the two-sided Spearman´s correlation coefficient showed a moderate correlation between HU values and RBC (*rho* = 0.390, *p* < 0.001) respectively F/P (*rho* = -0.384, *p* = 0.001). No significant correlation between HU values and WBC fraction, as well as between effective *Z* values and histological clot parameters were assessed, values are presented in Table 3.

**Table 3:** Correlation analysis of DE-CT parameters with histological clot composition

	RBC at histology (%)		F/P at histology (%)		WBC at histology (%)	
	*r*-value	*p*-value	*r*-value	*p*-value	*r*-value	*p*-value
RBC at histology (%)			-0.979	< 0.001	-0.254	0.034
F/P at histology (%)	-0.979	< 0.001			0.088	0.469
WBC at histology (%)	-0.254	0.034	0.088	0.469		
Attenuation (HU)	0.390	< 0.001	-0.384	< 0.01	-0.095	0.434
Effective *Z* value	-0.047	0.700	0.020	0.871	0.086	0.480

*F/P* Fibrin/platelets, *RBC* Red blood cells, *WBC* White blood cells

## Discussion

To the best of our knowledge, this is the first study to implement quantitative characterization of AIS clots *ex vivo* using DE-CT.

Using the multimaterial decomposition method with DE-CT, it is possible to determine the three main clot components, RBC, WBC, and F/P in comparison with histological analysis as a reference method [21]. Our correlation coefficients between clot composition of histological and DE-CT-based analysis resulted in the following data: RBC (*r* = 0.527, *p* < 0.001), WBC (*r* = 0.305, *p* = 0.020), and F/P (*r* = 0.525, *p* < 0.001). These results show the possibility to differentiate between RBC-rich and F/P-rich thrombi measuring clot attenuation in HU, as higher RBC fractions come along with higher HU than F/P-rich thrombi (51 *versus* 42, *p* = 0.005). In this study, arterioembolic and strokes of other determined causes summarized as non-cardioembolic showed more RBC and less F/P fractions than cardioembolic, as well as compared to cryptogenic. Comparison between the groups did not show a significant difference in the mean portions of WBCs.

DE-CT offers the opportunity to quantitatively determine highly mixed materials using information from two different energy levels [19]. In contrast to single-energy CT, DE-CT can differentiate between blood and iodinated contrast medium [23]. For AIS in particular, this allows differentiation between hyperdense areas after endovascular therapy, which may be either hemorrhage or contrast enhancement due to disruption of the blood–brain barrier [24, 25] and offers improved detection of acute ischemic lesions [26].

On conventional CT of patients with AIS, higher HU of *in situ* thrombi presented higher portions of RBC on subsequent histological analysis [3]. The presence of hyperdense artery sign suggests RBC-dominant or mixed clots and possibly predicts low F/P fractions [27]. This knowledge may have an impact on following treatment as intracranial thrombi with lower attenuation are associated with more difficult medical and interventional recanalization treatment [28] and longer intervention times [29]. In contrast, the hyperdense artery sign has been reported to be associated with more successful recanalization [30]. Experimental ovine blood clots with defined RBC amounts also differed in attenuation from fibrin-rich clots and showed higher HU in conventional CT [31]. In additional DE-CT imaging fibrin-rich clots presented an increase of attenuation after contrast medium exposure. Together with thrombus permeability on admission CT [18], these insights suggest that iodine uptake is dependent on F/P clot fraction.

In this study, the impact of histological composition on interventional treatment was not analyzed, but in further studies, especially RBC and F/P fractions seem to have an impact on interventional parameters and treatment success. Experimental fibrin-rich clots were associated with longer intervention time and lower recanalization rate than RBC-rich clots [11] and the amount of RBC seems to be associated with better reperfusion [12]. A per-pass analysis of histological clot composition showed that extracted thrombi showed higher RBC fractions within the first two passes than within further passes [32]. In summary, fibrin-rich thrombi are likely to be more resistant to recanalization treatment. Therefore, it is important to select appropriate mechanical devices depending on clot properties [14, 16]. CT imaging, especially DE-CT prior to intervention, may accomplish virtual histology of intracranial thrombi.

Furthermore, clot composition appears to depend on stroke pathogenesis. According to prior findings, arterioembolic clots and strokes of other determined causes are more likely to contain higher percentages of RBC [3, 6]. In contrast, cardioembolic and cryptogenic clots appear to be histologically similar, consisting of higher fractions of WBC and F/P [4–6]. Otherwise, certain studies exist that attained opposite conclusions and reported on higher fractions of F/P in large artery atherosclerosis or even higher RBC fractions in cardioembolic strokes than in large artery atherosclerosis [33, 34]. In particular, cryptogenic strokes are a challenge to relate histological clot composition to specific stroke causes, but similar histology suggests cardioembolic pathogenesis in most of these cases [4]. Indeed, there are studies that could not find associations between histological clot characteristics and stroke causes, and some even presented opposite findings [8].

This study has limitations. Thrombus material remaining *in situ* due to fragmentation or unsuccessful recanalization was not analyzed and important information may have been lost. Administration of the intravenous tissue plasminogen activator before intervention may also affect thrombus composition and fragmentation [35], but in this study the clot composition was determined *ex vivo* for both methods, using the DE-CT-based determination and histological analysis. For this reason, this limitation is important for the evaluation of the impact of histological clot composition on stroke pathogenesis. Additionally, formalin occupies an important fraction in multimaterial decomposition and x-ray mass attenuation coefficient of formalin cannot be neglected in DE-CT-based decomposition [21, 36], whereas in histological analysis no formalin is determined. The clots also showed a wide variation in storage time making it difficult to detect any changes in their composition. According to Douglas et al. [37], a change in clot composition of formalin-fixed clots is not expected if the analysis was performed within one week, which was done in this study.

In our case, the entire thrombus was segmented and the average absorption at high and low monoenergetic energy was used for evaluation. A resolution of finer structures and concentrations within the thrombi was not required for our question, as this was not relevant for the validation of the algorithm and for the comparison of different thrombi among each other. Should a future application of our methodology require a higher resolution to be able to represent concentration and structural differences within a thrombus, a photon-counting detector could potentially deliver successful results in this regard. If an even finer differentiation of the concentrations is to be made possible, there is also the possibility that measurements on a photon-counting detector could lead to success, as the spectral separation achieved by using energy thresholds could result in smaller concentration differences for this new question being better resolved. A frequently mentioned method for multimaterial decomposition is the so-called K-edge imaging, which is made possible using photon-counting technology [38]. However, it is important to point out at this point that this method is not suitable for use on thrombi, as it is only applicable if the K-edges of all materials lie in the medically relevant x-ray spectrum. This is not the case for our samples having a small nuclear charge number [39, 40]. Finally, the small sample size of this study should be considered. Stroke etiology was determined as arterioembolic in only six cases and as other determined cause in only five cases. Subsequently, only a small sample size of non-cardioembolic strokes was compared with cardioembolic and cryptogenic strokes. Statistical differences in histological clot composition between different stroke etiologies may not be reported.

In conclusion, DE-CT offers potential for multimaterial decomposition even in small objects as thrombus material and provides the ability to distinguish between RBC-rich and F/P-rich thrombi by measuring clot attenuation. Further studies should apply this method on patients who received DE-CT imaging prior to intervention to perform clot characterization *in situ*. These findings need to be correlated with interventional parameters to answer the question of whether it is possible to define personalized treatment strategies and to select between interventional devices according to clot composition in order to achieve faster reperfusion, higher recanalization rates, and better clinical outcomes.

## Data Availability

Data generated or analyzed during this study are available from the corresponding author by reasonable request.

## Abbreviations

AIS: Acute ischemic stroke
CT: Computed tomography
DE-CT: Dual-energy CT
F/P: Fibrin/platelets
RBC: Red blood cells
TOAST: Trial of ORG 10172 in Acute Stroke Treatment
WBC: White blood cells
